# Common FXIII and Fibrinogen Polymorphisms in Abdominal Aortic Aneurysms

**DOI:** 10.1371/journal.pone.0112407

**Published:** 2014-11-10

**Authors:** Fraser L. Macrae, Hannah Lee Evans, Katherine I. Bridge, Anne Johnson, D. Julian A. Scott, Robert A. S. Ariëns

**Affiliations:** 1 Theme Thrombosis, Division of Cardiovascular and Diabetes Research, Leeds institute for Cardiovascular and Metabolic Medicine, Multidisciplinary Cardiovascular Research Centre, University of Leeds, Leeds, United Kingdom; 2 Leeds Vascular Institute, The General Infirmary at Leeds, Leeds, United Kingdom; University of Bonn, Institut of experimental hematology and transfusion medicine, Germany

## Abstract

**Introduction:**

Abdominal aortic aneurysms (AAA) are characterized by a progressive dilatation of the abdominal aorta, and are associated with a high risk of rupture once the dilatation exceeds 55 mm in diameter. A large proportion of AAA develops an intraluminal thrombus, which contributes to hypoxia, inflammation and tissue degradation. We have previously shown that patients with AAA produce clots with altered structure which is more resistant to fibrinolysis. The aim of this study was to investigate genetic polymorphisms of FXIII and fibrinogen in AAA to identify how changes to these proteins may play a role in the development of AAA.

**Methods:**

Subjects of Western/European descent, ≥55 years of age (520 AAA patients and 449 controls) were genotyped for five polymorphisms (FXIII-A Val34Leu, FXIII-B His95Arg, FXIII-B Splice Variant (intron K nt29576C-G), Fib-A Thr312Ala and Fib-B Arg448Lys) by RT-PCR. Data were analysed by χ2 test and CubeX.

**Results:**

The FXIII-B Arg95 allele associated with AAA (Relative risk - 1.240, CI 1.093–1.407, P = 0.006). There was no association between FXIII-A Val34Leu, FXIII-B Splice Variant, Fib-A Thr312Ala or Fib-B Arg448Lys and AAA. FXIII-B His95Arg and FXIII-B Splice variant (intron K nt29576C-G) were in negative linkage disequilibrium (D’ = −0.609, p = 0.011).

**Discussion:**

The FXIII-B Arg95 variant is associated with an increased risk of AAA. These data suggest a possible role for FXIII in AAA pathogenesis.

## Introduction

Abdominal aortic aneurysms (AAA) are characterized by the progressive dilatation and thinning of the abdominal aortic wall which is secondary to chronic inflammation, matrix metalloproteinase (MMP)-mediated aortic wall destruction, degradation of elastin/collagen fibres and vascular smooth muscle cell apoptosis. [1]–[5] AAA commonly occurs in men over the age of 65 and accounts for up 1.8% of all deaths in England and Wales. [6], [7] An aneurysmal aorta has an anterior-posterior (inner to inner) diameter that is greater than 50% of the normal aortic diameter (2 cm) or greater than 3 cm. An AAA will continue to expand and will eventually rupture in the vast majority of cases when the anterior-posterior diameter exceeds 55 mm, leading to severe bleeding and a high risk of mortality. [8] The only current treatment option involves (open or endovascular) surgical intervention and the placement of a stent to prevent rupture; there are no drugs that can halt the disease process.

About 75% of all larger aneurysms are characterized by the presence of an intraluminal thrombus (ILT), which forms over many years, and persists even after endovascular repair when it remains present between the stent and the aneurysmal wall. [9], [10] The ILT is composed of a three dimensional (3D) fibrin network structure that incorporates red and white blood cells, platelets, other blood proteins, and cellular debris. Recent data show that the ILT increases biochemical stress on the aortic wall, leading to accelerated dilatation. [11] The thrombus is metabolically active and is constantly remodelled at the luminal surface. [11]–[13] The ILT is thought to attract leukocytes, which in turn release matrix metalloproteinases. [12] It also causes local hypoxia and leads to increased inflammation. [9] Furthermore, plasmin, which is generated on the fibrin surface, has been shown to play a role in aortic wall degradation through the activation of metalloproteinases 2 and 9. [14], [15] The degenerative changes and the ILT lead to focal aortic weakening, and due to a loss of structural integrity, the abdominal aortic wall is compromised. Little is known, however, about the pathogenesis of AAA rupture and what initiates it. In addition to the effects of the ILT, it has been found that patients with AAA show an increased risk of other cardiovascular disease [16] and demonstrate abnormal coagulation and clot structure profiles [6], [17]–[19].

The main proteins involved in thrombus formation are coagulation factors fibrinogen, thrombin and Factor XIII (FXIII). FXIII in plasma consists of two A-, and two B-subunits arranged as a heterotetramer. [20], [21] The A-subunits contain the active site of this protransglutaminase, whereas the B-subunits act as carrier molecules and are involved in FXIII activation through dissociation of the activated A-subunit dimer. [22] Recently, the interaction site for the A-subunit was shown to involve the second Sushi domain of the B-subunit between residues 96 and 103. [23] Fibrinogen is a hexamer**,** containing two sets of α-, β-, and γ-chains, linked to each other by 29 disulfide bonds. [24] Thrombin converts fibrinogen to fibrin, which polymerises and produces the protein network with which cells interact to form the blood clot. Activated FXIII cross-links fibrin to enhance its mechanical and proteolytic resistance. [22] Functional common genetic polymorphisms have been identified in both FXIII and Fibrinogen; these include FXIII-A Val34Leu [25]–[32], FXIII-B His95Arg [33], FXIII-B Splice Variant (intron K nt29576C-G) [34]–[36], Fib-α Thr312Ala [18], [37] and Fib-β Arg448Lys [19].

In view of the central role of FXIII and fibrinogen in thrombus formation and the complications of ILT in AAA, we investigated genetic polymorphisms of FXIII and Fibrinogen in AAA to identify high risk genotypes and potential disease mechanisms. We found that FXIII His95Arg associates with AAA supporting a role for FXIII in AAA pathogenesis.

## Methods

### Patient recruitment

Five hundred and twenty (n = 520) with an AAA and four hundred and ninety nine (n = 499) age-matched control subjects were enrolled as part of the Leeds Aneurysm Development Study (LEADS) with the inclusion and exclusion criteria previously described. [6], [38], [39] In brief, LEADS is a large, prospective, hospital based case-control study of AAA. Patients were recruited from the vascular outpatients department at the Leeds Teaching Hospitals NHS Trust (LTHT) in the north of the United Kingdom between 2003 and 2011. All subjects were of Western/European descent, aged ≥55 years and had an AAA ≥3 cm (defined as anterior-posterior diameter). Controls matched for age and ethnicity with an aortic diameter of 2.9 cm or less were recruited from family and friends of AAA patients from the vascular, general surgical and urology clinics in the same hospital trust. Survival data was obtained from hospital electronic patient databases. Ethical approval was obtained from the Leeds Teaching Hospitals research ethics committee (Ref: 03/142) and informed written consent was obtained from all participants.

### Blood sampling and DNA extraction

Six millilitres of free-flowing whole blood were collected from the antecubital vein into a K_2_EDTA coated blood collection tube, and stored at −20°C until required. DNA was extracted from whole blood using a QIAamp DNA Blood Maxi Kit (QIAGEN, Manchester, UK). The DNA samples were diluted to 10 ng/µl using DNA- and RNA- free Tris Buffered Saline, and stored at 4°C until genotyping. DNA concentration was measured using the 260/280 nm optical density ratio using a Nanodrop ND-1000 spectrophotometer (LabTech International, Uckfield, UK).

### Real Time Polymerase Chain Reaction

TaqMan single nucleotide polymorphism (SNP) Genotyping Assays (Life Technologies, Paisley, UK) were used to identify each of the polymorphisms (FXIII A Val34Leu (rs5985), FXIII B His95Arg (rs6003), Splice Variant (intron K nt29576C-G) (rs12134960), Fib-β Arg448Lys (rs4220) and Fib-α Thr312Ala (rs6050)). The PCR was set up in 384 well PCR plates with 2.2 µl of 10 ng/µl DNA in each well, 2.50 µl of Master Mix and 0.25 µl of probe. The TaqMan universal PCR master mix is a premix of all the components, except primers and probe, necessary to perform a 5′ nuclease assay. The final PCR conditions were: 95°C for ten minutes, 95°C for 15 seconds, and 60°C for one minute 30 seconds for 50 cycles. After PCR reactions were completed, the plates were cooled to room temperature and read in an ABI 7900 HT RT-PCR machine, and the results analysed by allelic discrimination of the sequence using detection software (Applied Biosystem, Life Technologies, Paisley, UK). Genotyping was called with an accuracy of 96–100%; samples which were not called successfully were repeated. Only samples that had all five genotypes successfully determined were included in the analysis.

### Fibrinogen Levels

Fibrinogen levels were measured using the Fibri-prest automate assay (Diagnostica Stago S.A.S, Asnières sur Seine, France) on a Start 4 coagulometer (Diagnostica Stago S.A.S, Asnières sur Seine, France). This assay is based on the clotting time of diluted plasma. This has a direct bearing on the level of plasma fibrinogen in the presence of excess thrombin. Polypropylene cuvettes were placed in the Start 4 coagulometer. A ball-bearing was placed into each cuvette which moved back and forth independently. 100 µl of plasma was added to each cuvette and heated to 37°C. Fibri-prest automate was added to each cuvette causing the plasma sample to clot. The ball bearing becomes enmeshed in the fibrin clot and therefore no longer moves independently. The clotting time (seconds) is taken from the addition of Fibri-prest automate to the time the ball bearing fails to move independently. This is inversely proportional to the amount of fibrinogen present (low clotting time = high fibrinogen).

### Statistical Analysis

Genotype distributions were tested for concordance with the Hardy-Weinberg equilibrium. Normality of continuous data was assessed using a one sample Kolmogorov-Smirnov test. Normally distributed continuous data was presented as the mean ± standard deviation and not-normally distributed data as the median (25^th^, 75^th^ percentiles). Independent Student *t* test was employed to test for differences between the means. Kaplan-Meier was used to assess overall survival. Genotype distributions and allele frequencies of each polymorphism were compared by the χ^2^ test. The Bonferroni correction was used to adjust for multiple testing. Linkage disequilibrium between polymorphisms was determined using ‘CubeX’, [40] and was expressed as D’. Data was analysed using SPSS v.20 (SPSS Inc. Chicago, Illinois, USA).

## Results

### Clinical characteristics

The demographic and clinical characteristics of the two groups, comprising of 520 AAA patients and 449 controls, are represented in table 1. As expected, the AAA group had larger aortic diameters compared with controls. There were higher rates of cardiovascular disease, including angina, cerebrovascular disease/transient ischaemic attacks, hypertension and myocardial infarction at recruitment, and a lower survival rate during follow up in the AAA group compared to controls, in agreement with previous studies. [41] There was a greater prevalence of current and ever smokers in the AAA group, in agreement with previous studies showing that smokers have a higher risk of developing a AAA compared with non-smokers. [42] A higher proportion of subjects were taking statins and aspirin in the AAA group, likely a reflection of the increased number of subjects with cardiovascular disease in this group. There was no major difference in alcohol consumption between the two groups. Fibrinogen levels were higher in patients with AAA compared with healthy control subjects (p = 0.0003).

**Table 1:** Demographic and clinical characteristics of the AAA and Control groups from the LEADS study.

	AAA (n = 520)	Control (n = 449)
**Age (years)^1^**	74.4 (7.5)	70.1 (7.3)
**Aortic Diameter (cm)^2^**	5.4 (4.0, 6.2)	1.9 (1.7, 2.3)
**Current smoker (%)^3^**	118 (27.8%)	73 (16.3%)
**Ever smokers (%)^3^**	473 (91.3%)	320 (71.3%)
**Alcohol, units/week^2^**	10 (4, 21)	10 (4, 18.5)
**CVD** * **^3^**	299 (57.5%)	207 (46.1%)
**Angina^3^**	140 (26.9%)	82 (18.3%)
**CVA/TIA^3^**	96 (18.46%)	60 (13.4%)
**MI^3^**	149 (28.7%)	50 (11.1%)
**Weight, Kg^2^**	78.9 (68.9, 89.0)	78.2 (70.0, 88.7)
**Hypertension^3^**	324 (62.6%)	199 (44.3%)
**Height, m^2^**	1.72 (1.67, 1.77)	1.70 (1.63, 1.75)
BMI**, Kg/m^2^^1^	27.1 (4.5)	27.6 (4.1)
**Waist: hip ratio^2^**	0.95 (0.90, 1.00)	0.93 (0.88, 0.97)
**Statins^3^**	370 (71.2%)	222 (49.4%)
**Aspirin^3^**	230 (44.2%)	137 (30.5%)
**Fibrinogen (g/L)^2^**	3.72 (3.19, 4.39)	3.51 (3.08, 3.51)

*Cardiovascular disease (CVD) = ischaemic heart disease (angina, MI or abnormal ECG) + peripheral arterial disease (PAD) + cerebro-vascular disease (TIA, stroke or known asymptomatic carotid disease). **BMI – body mass index.

1 - Parametric data expressed as mean (standard deviation).

2 - Nonparametric data expressed as median (25th, 75th quartiles).

3 - Categorical data expressed as No. (%).

Genotype distributions for each polymorphism were consistent with the Hardy-Weinberg equilibrium (table 2), and overall allele frequencies were consistent with other studies on these polymorphisms in different cardiovascular diseases [18], [36], [43], [44].

**Table 2:** Allelic frequencies of FXIII and fibrinogen gene polymorphisms compared with Hardy-Weinberg equilibrium.

		Total population	
	Rare Allele Frequency	X^2^	P
**FXIII-A Val34Leu**	0.26	2.199536	0.138052
**FXIII-B His95Arg**	0.11	1.260245	0.261605
**FXIII-B splice variant**	0.17	0.336225	0.562016
**Fib-α Thr312Ala**	0.25	2.887291	0.089281
**Fib-β Arg448Lys**	0.18	0.010879	0.916928

### Polymorphic allele distribution in AAA vs Controls

The distribution of the FXIII-B 95Arg allele was significantly different between AAA patients and controls, with the percentage of subjects heterozygous and homozygous for the variant 1.45 times and 2.33 times higher in the AAA group respectively when compared with controls (p = 0.006; p = 0.03 after Bonferroni adjustment for multiple testing) (table3). When combining the heterozygote and homozygote carriers of the rare allele, the relative risk of developing an AAA with the FXIII-B 95Arg allele was 1.240 (CI 1.093–1.407). There were no associations between the rare alleles of FXIII-A Val34Leu, FXIII-B Splice variant, Fib-α Thr312Ala or Fib-β Arg448Lys polymorphisms and AAA (Table 3).

**Table 3:** Polymorphism distribution in AAA vs Controls.

Polymorphisms		AAA (n = 520)	Control (n = 449)	P
**FXIII-A Val34Leu**	**Val/Val**	56.3% (293)	57.9% (260)	0.713
	**Val/Leu**	35.8% (186)	33.4% (150)	
	**Leu/Leu**	7.9% (41)	8.7% (39)	
**FXIII-B His95Arg**	**His/His**	76.0% (395)	84.0% (377)	0.006
	**His/Arg**	21.9% (114)	15.1% (68)	
	**Arg/Arg**	2.1% (11)	0.9% (4)	
**FXIII-B Splice Variant**	**Wt/Wt**	68.3% (355)	69.7% (313)	0.890
	**Wt/SV**	28.5% (148)	27.2% (122)	
	**SV/SV**	3.3% (17)	3.1% (14)	
**Fib-α Thr312Ala**	**Thr/Thr**	56.7% (295)	53.2% (239)	0.331
	**Thr/Ala**	38.8% (202)	40.5 (182)%	
	**Ala/Ala**	4.4% (23)	6.2% (28)	
**Fib-β Arg448Lys**	**Arg/Arg**	65.2% (339)	68.6% (308)	0.355
	**Arg/Lys**	30.8% (160)	28.7% (129)	
	**Lys/Lys**	4.0% (21)	2.7% (12)	

Data expressed as percentage (No.), analysed by X^2^.

### Association of polymorphisms with fibrinogen levels

Patients who possessed at least one Fib-β 448Lys allele (3.81 g/L) were found to have significantly higher fibrinogen levels than patients homozygous for the Arg allele (3.55 g/L) in the whole study population (p = 0.001) (Table 4). Fibrinogen levels were found to increase step wise with an increase in the number of Lys alleles, Arg/Arg 3.55 g/L (3.08,4.13), Arg/Lys 3.74 g/L (3.22, 4.44) and Lys/Lys 4.18 g/L (3.41, 4.53). There was no association of fibrinogen levels with FXIII-A Val34Leu, FXIII-B His95Arg, FXIII-B Splice variant and Fib-α Thr312Ala polymorphisms (Table 4).

**Table 4:** Association of polymorphisms with fibrinogen levels in total study population.

Polymorphisms		Fibrinogen levels g/L	P
**FXIII-A Val34Leu**	**Leu −**	3.56 (3.11, 4.23)	0.313
	**Leu +**	3.67 (3.19,4.24)	
**FXIII-B His95Arg**	**Arg −**	3.59 (3.12, 4.24)	0.136
	**Arg +**	3.76 (3.26, 4.25)	
**FXIII-B Splice Variant**	**SV −**	3.66 (3.16, 4.26)	0.318
	**SV +**	3.55 (3.10, 4.19)	
**Fib-α Thr312Ala**	**Ala −**	3.62 (3.19, 4.22)	0.526
	**Ala +**	3.62 (3.06, 4.27)	
**Fib-β Arg448Lys**	**Lys −**	3.55 (3.08, 4.13)	0.001*
	**Lys +**	3.81 (3.23, 4.46)	

Data expressed as Median (25^th^, 75^th^ quartiles), analysed by Mann-Whitney U.

### Association of Alleles

In the total study population there was evidence of negative linkage disequilibrium between the His95Arg and Splice Variant polymorphisms (D’ = −0.609, P = 0.011) (Table 5). In the control subjects the polymorphisms were found to be close to complete negative disequilibrium (D’ = −1.0, P = 0.018). The association of His95Arg with AAA was still significant if subjects with the Splice Variant were excluded from the analysis (P = 0.016).

**Table 5:** The association between the FXII-B His95Arg and Splice Variant polymorphisms in the total study population.

			FXIII-B Splice Variant			
		Wt/Wt	Wt/SV	SV/SV	Total	P
	**His/His**	511	233	28	772	0.011
		(52.7)	(24)	(2.9)		
**FXIII-B His95Arg**	**His/Arg**	145	34	3	182	
		(15)	(3.5)	(0.3)		
	**Arg/Arg**	12	3	0	15	
		(1.2)	(0.3)	(0)		
	**Total**	669	270	31	969	

Wt – wild type, SV-splice variant, Data expressed as No. (%), analysed by X^2^.

## Discussion

In this study we investigated the link between five previously described polymorphisms in the FXIII and fibrinogen genes, and AAAs. The polymorphisms we investigated were FXIII-A Val34Leu, FXIII-B His95Arg, FXIII-B Splice Variant (intron K nt29576C-G), Fib-A Thr312Ala and Fib-B Arg448Lys. We found that there is an association between the Arg allele at position 95 of FXIII-B and the occurrence of AAA. We also showed that there was no association between any of the Fibrinogen polymorphisms and AAA. Finally we showed that FXIII-B His95Arg and FXIII-B splice variant were in negative linkage disequilibrium.

There was an over-representation of the Arg allele in patients with AAA suggesting that FXIII-B Arg allele is a potential risk factor for the development or progression of an AAA. The FXIII-B His95Arg polymorphism has previously been linked to a 50% increased risk of venous thrombosis and has been shown to increase subunit dissociation of the FXIII-A and -B subunits in plasma. [33] Recently it has been shown that in the N-terminal region of FXIII-B, within the peptide YGCASGYK spanning positions 96–103 in the second sushi domain, there is an interaction site for FXIII-B with FXIII-A_2_. [23] FXIII-B His95Arg occurs in the amino acid position immediately before the start of this interaction site and may play a role in FXIII-A_2_ and FXIII-B interactions. Substitution of His to Arg at position 95 could therefore have an effect on FXIII activation which could result in altered cross-linking activity, potentially influencing the stability of the ILT. An increase in fibrin clot stability likely leads to a more stable, lysis resistant ILT, which in turn may enhance the inflammatory and proteolytic insult on the underlying aortic wall, resulting in AAA progression. Although the B-subunit has no enzymatic activity, it has been suggested that FXIII-B may help to stabilize FXIII-A in plasma and also be important in the secretion of the FXIII-A subunit from its site of synthesis in the bone marrow. [34] In addition, there may be hitherto unknown functional roles for FXIII-B after it has dissociated from FXIII-A. As FXIII-B may bind to fibrinogen, [45] FXIII-B may also play an important role directing FXIII transglutaminase activity towards its main substrates fibrin. Our findings indicate that further studies are warranted to fully investigate the role of FXIII in AAA.

Alternatively, the FXIII His95Arg polymorphism could be affecting the role of FXIII in tissue repair of the diseased aortic wall. The FXIII A-subunit belongs to the transglutaminase family of enzymes, which are known to cross-link various matrix proteins and are involved in tissue repair. [46] Indeed, FXIII may cross-link collagen and fibronectin for example, two proteins involved in the extracellular matrix of the vascular wall. Due to its effect on increased subunit dissociation, the FXIII-B His95Arg polymorphism could increase FXIII activation at the site of fibrin deposition and within the ILT, reducing the amount of FXIII available for diffusion into the aortic wall. An increase in the stability of the ILT would result in a thicker ILT, so that local hypoxia of the underlying wall would be more marked, and there would also be a larger distance between the lumen and the wall, so repair proteins would have further to travel. This could negatively affect the role of FXIII in repairing the tissue damage that occurs in the aortic wall, resulting in an increased level of AAA development. In support of a systemic role of FXIII in arterial wall repair FXIII V34L has been previously associated with arachnoid aneurysms [47].

Genetic association studies on multiple SNPs can result in type I errors, when the null hypothesis is rejected due to multiple testing, but there is no true association between the polymorphism and the phenotype. [48] To correct for this we used the Bonferroni adjustment for multiple testing. After multiplication of the P values by the total number of SNP’s tested, the *P* value for the association of FXIII-B His95Arg and AAA was 0.03 so should still be considered significant. However, our findings will need to be confirmed in other, large studies of FXIII in AAA.

There is an ongoing debate in previous literature whether Fib-β Arg448Lys is associated with a change in fibrinogen levels. Some studies found no effect on fibrinogen concentrations [49] and some have found that Fib-β 448Lys was associated with an increase in fibrinogen concentrations. [50] We demonstrated increased fibrinogen levels in patients possessing the Fib-β 448Lys allele, and the association appeared to be related to the number of Lys alleles which is largely in agreement with the observations by Kain et al, 2002. [50] Fib-β Arg448Lys has been shown to be in linkage disequilibrium with fib-β -455 G/A [44], [50], and fib-β -455 G/A has also been associated with an increase in fibrinogen levels. [51] The inconsistency of the relationship between Fib-β Arg448Lys and fibrinogen levels is likely a reflection of complex gene-environment interactions including several genetic and environmental factors. FXIII-B His95Arg was not found to be associated with fibrinogen levels, so its association with AAA is not due to fibrinogen levels.

We found a strong negative linkage disequilibrium between the His95Arg and Splice Variant polymorphisms (D’ = −0.609) of FXIII, and in the control subjects the polymorphisms were found to be closer to complete negative disequilibrium (D’ = −1.0). This should be interpreted cautiously, as this relationship may be due to low numbers of heterozygotes for both polymorphisms. Our findings indicate that the minor allele at one locus is associated with the major allele at the other locus. Both of these polymorphisms have previously been reported to show considerable geographic differentiation, with FXIII-B His95Arg being most common in populations of African descent, whereas the FXIII-B splice variant is most common in populations of Asian descent. [36] This could help to explain the low numbers of heterozygotes and homozygotes for the rare allele for both polymorphisms. Although the two factor XIII polymorphisms were in strong negative linkage disequilibrium, the association of His96Arg with AAA was not observed with the Splice Variant. The association of His95Arg with AAA was still observed when we only included subjects who do not possess the splice variant in the analysis. This further corroborates that the FXIII His95Arg substitution plays a role in AAA and that its effects do not appear to occur through linkage disequilibrium with the FXIII-B splice variant. However, linkage disequilibrium with other potentially functional variants cannot be excluded with our data, and future studies, including genome wide association studies, are required to confirm these findings.

In conclusion, we have found that FXIII-B Arg95 is associated with increased risk of developing AAA. FXIII-B His95Arg was shown to be in negative disequilibrium with the FXIII-B Splice Variant. Further studies are warranted to elucidate the role of FXIII in AAA pathogenesis using *in vitro* and *in vivo* models to clarify the underlying mechanisms and provide potential avenues in the development of new therapeutic agents.
